# A Compiled Update on Nutrition, Phytochemicals, Processing Effects, Analytical Testing and Health Effects of *Chenopodium album*: A Non-Conventional Edible Plant (NCEP)

**DOI:** 10.3390/molecules28134902

**Published:** 2023-06-21

**Authors:** Sukhwinder Singh, Amandeep Singh, Supandeep Singh Hallan, Agnese Brangule, Bhupinder Kumar, Rohit Bhatia

**Affiliations:** 1 Department of Pharmaceutical Analysis, ISF College of Pharmacy, Moga 142001, Punjab, India; sukhwinde932@gmail.com; 2 Department of Pharmaceutics, ISF College of Pharmacy, Moga 142001, Punjab, India; ad4singh@gmail.com; 3 Department of Pharmaceutical Chemistry, Riga Stradins University, Konsula 21, LV-1007 Riga, Latvia; supandeepshallan@gmail.com (S.S.H.); agnese.brangule@rsu.lv (A.B.); 4 Baltic Biomaterials Centre of Excellence, Headquarters at Riga Technical University, Kalku Street 1, LV-1658 Riga, Latvia; 5 Department of Pharmaceutical Sciences, HNB Garhwal University, Chauras Campus, Srinagar 246174, Uttarakhand, India; bhupinderkumar25@gmail.com; 6 Department of Chemistry, Graphic Era (Deemed to be University), Dehradun 248002, Uttarakhand, India

**Keywords:** *Chenopodium album*, food applications, impact of the processing, ethnobotanical use, non-conventional edible plants, ascaridole

## Abstract

Bathua (*Chenopodium album*) is a rich source of extensive-ranging nutrients, including bio-active carbohydrates, flavonoids and phenolics, minerals, and vitamins that translate to countless health benefits such as anticancer, antidiabetic, anti-inflammatory, antimicrobial, and antioxidant activity. Ascaridole, an important phytoconstituent present in aerial parts of the plant, contributes to its anthelmintic property. Even with vast historical use and significant health benefits, its renown has not spread, and utilization has significantly decreased in recent decades. Gradually, the plant has become known under the name of Non-conventional edible plant (NCEP). This compilation is prepared to bring out the plant under the spotlight for further research by foregrounding previous studies on the plant. Scientific research databases, including PubMed, Google Scholar, Scopus, SpringerLink, ScienceDirect, and Wiley Online, were used to fetch data on *C. album*. This review offers over up-to-date knowledge on nutritious values, phytochemical composition, volatile compounds, as well as health benefits of *C. album*. The ethnobotanical and ethnomedicinal uses of the plant in India and other parts of the world are deliberately discussed. Scrutinizing the reported literature on *C. album* reveals its powerful nutrient composition advantageous in the development of food products. The impact of various cooking and processing methods on the nutritional profile and bioavailability are discussed. The future perspectives with regards to the potential for food and nutraceutical products are critically addressed. This review proves the necessity of breakthrough research to investigate the pharmacology and safety of phytochemicals and nutraceutical development studies on the *C. album*.

## 1. Introduction

Mother nature has been a bottomless resource for fulfillment of mankind’s needs. Plants have been used traditionally for hunting craftworks, beautification, food preparation, furniture, and medicinal uses including emergency treatments. Although 27,000 plant species are estimated in the world to have food potential, an estimated 103 species have been reported to contribute to the 90% of the world’s food supply [1,2,3,4,5]. Possessing valuable nutritional and sensory characteristics, numerous edible plants which are locally grown remain untamed. These plants are referred to as Non-conventional edible plants (NCEP). These seasonal and regional plants have food potential, but their use is not widespread due to limited or non-existent research. Nevertheless, their immense and indispensable value has driven a gradually increasing scientific interest in these plants. The consumption of NCEPs could be a virtuous way to contribute to sustainable food systems [6,7].

*Chenopodium album Linn.* (Fat hen, lamb’s quarter), Amaranthaceae, has a sizeable background of traditional use in India and other parts of the world. The plant has been consumed as an important food source since antiquity, with leaves and tender twigs consumed as vegetables [8,9]. *C. album* has been utilized for several disease-modifying and health-promoting activities in the cardiovascular, circulatory, digestive, and immune systems. The plant grows low in height relative to almost every winter-cultivated crop of the tropical and subtropical regions, including barley, gram, mustard, and wheat [10]. The weed is often allowed to grow with the cultivated crops in Northern India due to its excellent composition of nutritional components [11].

Interestingly, the plant leaves have been utilized for different purposes; they are integrated with food and fodder. The leaves and tender shoots are consumed in raw form as a salad or cooked into vegetables. The dough prepared by mixing *C. album* leaves with wheat flour is often cooked to paratha (resembling flatbread) for eating [12]. Although the plant is neglected and underutilized, it has been considered the future of smart food [13]. Apart from conventional use in food, the plant has been used to cure different diseases. Juice of leaves is used for the treatment of burns, while the powdered leaves are often dusted on the irritating skin surface. Moreover, the aerial parts of the plant and their decoction are used to rub on the affected body parts to achieve relief from rheumatism and arthritis in the bygone times [14,15]. The plant is advantageous to use in dyspepsia, flatulence, intestinal and peptic ulcer, ophthalmopathy, splenopathy, and strangury [16]. The oil made from the plant has been employed for paralyzing or killing intestinal worms. Additionally, the plant has historical use for analgesic, anti-inflammatory, diuretic, and sedative activity [17]. The plant is used as a blood purifier in folk medicine withal.

The high protein content and a balanced spectrum of amino acids are some of the fascinating features of the plant; leucine, lysine, and isoleucine are the predominant amino acids present in the leaves. The vitamin and mineral composition of the plant includes retinoic acid, ascorbic acid, thiamine, riboflavin, niacin, potassium, magnesium, calcium, iron, phosphorous, and traces of pantothenic acid [11]. The chief phytochemical compounds reported in the plant belong to the class of alkaloids, flavonoids, steroids, and saponins. In addition to the above, the oil obtained by hydro-distillation of leaves has been found to contain *p*-cymene and the anthelmintic compound ascaridole [18].

Although the plant is consumed in the form of cooked vegetables as well as a salad in food, its use is not widespread. The manual cultivation of the plant is generally not practiced due to its self-pollinating nature [19]. In addition, the inhibited growth of plants due to the added weedicides in the crops is leading to reduced plant availability in the market. Unfortunately, the interest and knowledge of the plant have significantly decreased in the recent generations.

Despite its traditional use since historical times, the plant has not undergone any research studies of renowned character, which represents the need for intensive exploration of the plant. Recent research has focused on exploring the phytochemicals of the plant [20,21]. Also, the pharmacological activity of the plant extract toward several bacterial and viral organisms has been determined, but the determination of the nutraceutical and food potential of the plant has not gained much attention [17,22]. In recent years, S. Singh has focused on the physicochemical, textural, sensorial, and nutritional characteristics of the plant [23,24,25]. However, its potential of the plant is not fully explored yet. This review compiles the recent literature reporting on the nutritional potential, food potential, and safety of *C. album*. Moreover, the ethnobotanical and ethnomedicinal use of the plant highlighting its possible medicinal importance in modern medicine is discussed. This review can serve as an updated compendium of thorough insights of *C. album* in relation to its botanical and cultivation characteristics, ethnomedicinal use, nutritional and phytochemical composition and impact of different processing methods on the same, analytical methodologies so far employed for the analysis, safety, and food potential of the plant that would help researchers to further augment the research on the plant.

## 2. Characteristics and Cultivation of *C. album*

### 2.1. Botanical Description and Vernacular Names

*C. album* is a pale green, erect, and strong-smelling polymorphous annual herb of a height of up to 3.5 m, growing 3600 m above sea level. The word “Chenopodium” is derived from two Greek words, viz., “khen” (goose) and “pous” (foot), describing the goosefoot shape of most of the species belonging to the genus. The taxonomic features of the plant include kingdom, phylum, subphylum, class, order, and family, viz., Plantae, Spermatophyta, Angiospermae, Dicotyledonae, Caryophyllales, and Amaranthaceae. The leaves are simple and alternate, oval to obovate or lanceolate shaped with length and width of 1.5–8 cm and 3 cm, respectively, and are attached to the petiole. Decussate leaves are grown first, followed by alternating with long, oval onboard teethed alternate leaves. The stem is cylindrical to angular, erect, grooved, usually branched, and often reddish [26,27]. Dense inflorescence formed by the aggregation of flowers are attached to the leaf axils at the terminus of stems. Small flowers possess radial symmetry and grow on dense branched inflorescence of 10–40 cm in length. The seeds are black, smooth and shiny, horizontally flattened, and lenticular with a diameter of 0.7–1.5 mm. The cotyledons are fleshy, elliptic, elongated, 10–15 mm long and 2–3 mm wide, and shortly petiolate [28,29].

The common English names of *C. album* include “Melde”, “Lamb’s quarters”, Fat-hen”, “Goosefoot”, “Misbredie”, “Withondebossie”, “Umbikicane”, “Bloubossie”, “Wild spinach”, “Misbredie”, “Varkbossie” and “Pigweed”. The vernacular names used in different parts of India are “Chandan betu” (Bengali), “Pappukura” (Telgu), “Bathua Sag” (Hindi), “Katu ayamoddakam” (Malayalam), “Parupukkirai” (Tamil), “Bathava” (Gujrati) and “Bathu” [15]. The plant names according to Ayurveda, Siddha, Folk and Unani systems of medicine are “Vaastuuka”, “Paruppukeerai”, “Chilli-shaak”, and “Baathu”, respectively [9].

### 2.2. Cultivation Information

*C. album* is a widely distributed weed plant, mainly in Asia, Africa, Europe, and North America; it grows in nitrogen-rich soils. Around 21 species of the plant are found in India, particularly in Rajasthan, Kullu valley, and Shimla. The most likely companions of the weed plant are corn, potatoes, and cucurbits. The plant can be traditionally grown in nitrogen-rich soils using the hydroponic method [30,31]. The soil required for the growth of the plant should be moderately fertile; a pH of 4.5 to 8.3 is tolerable. The germination potential of freshly harvested seeds remains around 35%; however, low-temperature (0 to 5 °C) treatments have been found to increase the germinability as prolonged soaking over 20 days do. The percentage of germination is maximum for seeds lying just below the soil surface. The difference in germination optima at different temperatures reflects the different behavior of plants at varying places [26]. Interestingly, the plant is frost tolerant. The longer exposure of the plant to sunlight results in larger and more vigorous plants which discloses the reason for sparse distribution around the equator and extensive distribution in temperate regions [32].

## 3. Ethnobotanical and Ethnomedicinal Use of *C. album*

The traditional ayurvedic book *Ashtang Hridaya* emphasizes the importance of meshed dishes prepared by cooking greens of *C. album*. The ancient “Vedas” written by sages also puts the plant into the spotlight. The “Rig Veda” and “Atharva Veda” highlight the beneficial effects of *C. album* in the treatment of piles, clearing worms, and as a laxative. The knowledge compiled from these Vedas and edited by “Agnivesha” and “Charaka”, respectively, has led to a legendary compilation, *Charaka Samhita*, which, along with *Sushruta Samhita*, is still used by the practitioners of the traditional system of medicine. Both these books underscore the importance of *C. album* in improving digestive power, memory, appetite, and body strength [33,34,35]. In addition, it has been said to have purgative action and help to relieve constipation [36].

Although manual cultivation of the herbaceous pot plant, commonly known as *bathua,* is not widely practiced in India, its growth can be easily detected in the corners of early grain fields in the country. Since long ago, the plant has been employed in the diet as well as for the management of several diseases; the leaf extract is still used in the Ladakh region (India) for controlling painful urination [37]. Ethnomedicinal surveys revealed the utilization of decoctions prepared from different plant parts as herbal remedies for several diseases. Whole-plant decoction has been employed for anthelmintic purposes and the management of jaundice and other liver diseases in various parts of Pakistan (PAK), including Hattar, Gulla Khel, and Makerwal [38,39]. Additionally, the decoction prepared from aerial parts is known to be utilized to treat stomach diseases and gastrointestinal disorders in Gilgit-Baltistan, PAK [40]. The use of the plant for treating indigestion and constipation is also practiced in the Parbati Valley of Kullu, and Sikandra Hill Range of Mandi, Himachal Pradesh [41,42]. In addition, the anthelmintic property has also been reported in myrrh (*Commiphora molmol*), tulsi (*Ocimum sanctum*), papaya (*Carica papaya*), and ginkgo (*Ginkgo biloba*) [43].

Moreover, the treatment of kidney stones and urinary tract complications with cooked *C. album* leaves and/or herbal tea made from them is considered to be the appropriate treatment option in the folk medicine of Rajasthan, Toba Tek Singh, and Azad Jammu and Kashmir [44,45,46]. Additionally, people of the Shekhavati region of Rajasthan make use of cooked *C. album* leaves for the treatment of colic and other urinary system issues [47]. Furthermore, the tribal use of fresh leaves and flowers for vegetables and dried plant powder for diuretic purposes has been reported in Chonthra Karak (Pakistan), and Garhwal (India) [48].

Interestingly, the plant is reported to possess sexual health-promoting properties [49]. The oral consumption of whole-plant powder for the treatment of sexually related problems is practiced in Gujranwala and Lower Kurram [50,51]. In the trans-Himalayan region of India, half a spoon of whole-plant powder is used for treating headaches and seminal weakness [52]. The use of plant seeds and leaves for the treatment of unconsciousness and removal of thirst has also been reported in Mirpur (Pakistan) [53]. Interestingly, the use of amla (*Emblica officinalis*), black pepper (*Piper nigrum*), wild mint (*Mentha arvensis*), tamarind (*Tamarindus indica*), and allium (Allium odorosum) has also been reported for the treatment of kidney stones by Muslim herbalists [54].

The ethnobotanical uses of cooked plant parts (leaves and stems) further extend to the treatment of flu, gall stones, and tuberculosis. The traditional uses of the plant also include the treatment of sunstroke, sunburn, and swollen feet [55,56]. The herbal drink (fresh infusion) prepared from the whole plant is also used to treat intestinal ulceration in traditional communities of Pakistan [57]. Besides the other uses of the plant, the village peoples of Thoppampatti, Tamilnadu utilize the whole plant for anti-scorbutic uses [58]. Furthermore, the plant is also used as a blood purifier by rural communities of the Arid regions of Punjab, Pakistan [59]. The plant is used for treating skin-related problems in Dehradun, Uttarakhand [60]. The use of the whole plant in the treatment of enlarged spleen and plant roots for the treatment of rheumatism and snake poison in Islamabad is also being practiced [61]. Additionally, the erythropoiesis-stimulating activity of the *bathua* plant is ethnobotanically employed for the treatment of anemia in the Kumaun Himalayan region [62].

## 4. Nutritional and Phytochemical Profile of *C. album*

### 4.1. Vitamins and Minerals

The plant is a rich source of vital minerals and vitamins for the body in which retinol, ascorbic acid, B-complex, calcium, and potassium are predominant. The mineral content present in the leaves of *C. album* is much comparably higher than in other consumed vegetables such as beet, mustard leaves and spinach [63]. The concentration of minerals varies among raw and cooked vegetables. The average content of minerals/100 g of raw lamb’s quarters is known to be as follows: calcium—309 mg, magnesium—34 mg, potassium—452 mg, iron—1.2 mg, phosphorous—72 mg, sodium—43 mg, and other elements including selenium, copper, manganese, and zinc in small (less than 1 mg) quantities. Zinc and iron present in leafy vegetables are essential for a healthy immune system and combating anemias. In addition, the plant is a good reserve of retinol and ascorbic acid. The average vitamin content present/100 g of the raw plant is noted to be the following: retinoic acid—11,600 IU, ascorbic acid—80 mg, niacin—1.2 mg, and a trace amount of thiamin, riboflavin, pantothenic acid, pyridoxal, and folate (30 µg/100 g) [64]. One study utilized the thiocyanate method to determine in vitro bioavailability of iron from fresh and dehydrated leaves of Bathua and food made from it [65]. The in vitro bioavailability of iron from paratha and laddoo made from leaves was found to be 2.16 mg/100 g and 2.78 mg/100 g. In addition, the calcium present in raw as well as cooked *C. album* leaves has been reported to be 32 to 33% bioavailable [66].

### 4.2. Carbohydrates

Polysaccharides are formed by the linking of several monosaccharide units attached by glycosidic linkages. Further, the biological functions of the resulting polymers are influenced by the degree of polymerization, types of monosaccharide units attached, and the glycosidic linkages. The total carbohydrate content in the raw and cooked lamb quarters is reported to be 7.3 g and 5 g/100 g, respectively [64]. Fructose, glucose, lactose, maltose, and sucrose have been reported in young shoots and mature plants [67]. Dietary fiber is part of a plant-derived food product that is not completely digestible in the human intestines. The consumption of a fiber-rich diet is associated with a reduced risk of cardiovascular diseases. Total dietary fibers represent about 4 g of total carbohydrates in 100 g of raw vegetables. However, some studies have reported a higher content of dietary fibers in young shoots and mature plant material [63].

### 4.3. Protein and Amino Acids

The nutritional quality of a plant in terms of protein content is governed by the presence and proportion of essential amino acids in the plant/food product. Essential amino acids are not biosynthesized by the human body and are vital for bodily functions. High protein content and a balanced spectrum of amino acids are some of the reasons behind the consumption of *C. album*. The green leafy part and seeds represent the highly valuable parts for the protein content, particularly higher in lysine due to its synthesis and gathering in a soluble and protein form. The average protein content in the raw lamb’s quarters is known to be 4.2 g/100 g of material [64]. However, some studies have reported even higher protein content, such as 203 g/kg and 32.2 g/100 g in vegetation matter [67,68]. The content of isoleucine, leucine, phenylalanine, threonine, and valine in the green matter protein is reported to be even higher than that in seeds [15]. Among essential amino acids, arginine (11.29 g/kg), leucine (13.44 g/kg), lysine (10.11 g/kg), and phenylalanine (9.26 g/kg) are mainly present in the green matter, while those predominating in seeds are arginine (17.18 g/kg), leucine (7.58 g/kg) and lysine (8.07 g/kg) [68].

### 4.4. Fatty Acids

Quite a low yield of oils but rich amounts of essential oils are found in the leaves of wild edible plants. Essential fatty acids belonging to the ω^3^ series were found in the *C. album*. These fatty acids are known to play a valuable role in modulating human metabolism as well as prevent against coronary heart diseases [69,70]. According to the U.S. Department of Agriculture, the total lipid content in the raw lamb’s quarters is reported to be 0.8 g/100 g, with saturated fatty acids (0.059 g), monounsaturated fatty acids (1.05 g), and polyunsaturated fatty acids (0.351 g) [64]. The predominant fatty acids are 18:3ω^3^, 18:2ω^6^, and 16:0. However, unusual fatty acids such as eicosapentaenoic acid and docosahexaenoic acid are absent in the leaves [70]. The polyunsaturated fatty acid composition of a plant may provide beneficial effects towards prevention of diseases such as osteoarthritis and autoimmune disorders. Comparatively, the fatty acid content in leaves is higher compared to that of roots. Along with the salt tolerance of *C. album*, the total composition of fatty acids in the plant parts was reported to remain unaffected under salt stress conditions [21].

### 4.5. Phytochemicals

Apart from the appreciable composition of carbohydrates, protein, and fats, other phytochemicals belonging to the class of alkaloids, saponins, terpenoids, flavonoids, and phenolic compounds make *C. album* a versatile revitalizing source. Phenolic compounds in the plant are responsible for several biological activities, including anticancer, antihyperglycemic, anti-inflammatory, antimicrobial, and lowering of adipogenesis [9,22,71]. The high content of crude alkaloids is responsible for the spasmolytic and anesthetic activity. Additionally, the leaves are reported to contain carotenoids which possess provitamin-A activity and hence promise antioxidant activity [72].

The essential oil obtained by hydro-distillation was found to be composed of higher hydrocarbons, oxygenated and bicyclic mono-, di- and sesquiterpenoids, and fatty acids [22]. Volatile oil obtained from the plant was found to be composed of (*E*)-ascaridole, carvacrol, (*Z*)-carvyl acetate, *p*-cimen-80-ol, (*E*)-piperitol acetate, benzyl alcohol, *p*-mentha-1,3,8,-triene, *p*-cymene, α-terpinene, *p*-cresol, and piperitone [73]. These components were reported to account for 90.4% of total volatile oil. Phytol was identified to be the most oxygenated diterpene in the oil. In addition, some studies have reported the α-pinene as the most abundant and pinane-2-ol to be the most oxygenated monoterpene component of the essential oil [18].

**Figure 1:**
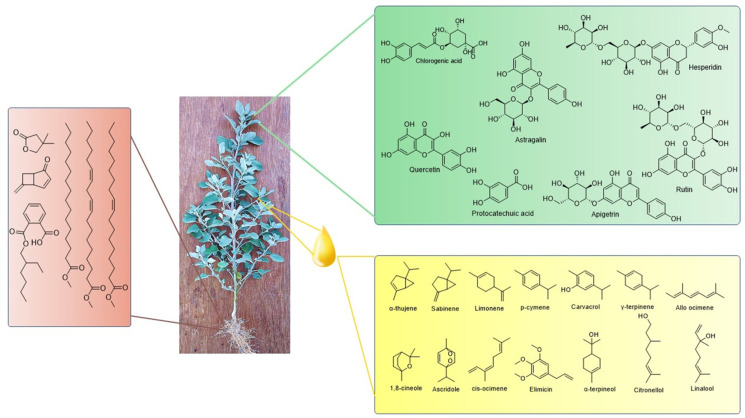
Biologically active phytoconstituents present in different parts of *C. album*.

Furthermore, ester compounds of hexadecenoic acid, 9-Octadecenoic acid and 9,12-Octadecadienoic acid owing to valuable activities such as antibacterial, antifungal, antioxidant, antiviral, anti-inflammatory, and nematocidal activity have been reported to be present in the roots of the plant [74,75]. Volatile organic compounds synthesized by microorganisms have shown significant plant growth-promoting activity by regulating photosynthesis and other vital functions. Interestingly, the volatile organic compound cryptomeridiol obtained from seeds shows significant plant growth-promoting activity [9,76,77]. The methanolic extract of roots has revealed the presence of a novel compound, chenoalbicin, with an alkaloid moiety attached to a cinnamic acid amide. The compound was reported to have allelopathic effects [78,79]. The chemical structures of phytochemicals identified in the different plant extracts and oil are given in Figure 1. Phytic acid, an antinutritional compound present in the leaves of the plant, acts as a major phosphorous storage compound. The phytochemical impairs the absorption of important mineral ions such as calcium, iron, and zinc, which are present in a co-administered diet [80].

## 5. Extraction, Isolation, and Analysis of Bioactive Phytochemicals from *C. album*

The separation of bioactive phytoconstituents such as polysaccharides, phenolic compounds, and proteins from the plant material by employing standard procedures and specific composition of solvents is termed “extraction”. The extraction efficiency for a particular type of compound differs for extraction methods [81]. The type of extraction method to be employed depends on the plant part as well as the type of phytoconstituents to be analyzed. Soxhlation, solvent extraction, maceration, and hydro-distillation are the widely employed methods for phytochemical extraction from *C. album*. Additionally, microwave- and ultrasonic-assisted extraction can also be employed for the phenolic content analysis of food samples.

Following extraction, the foremost step in the analysis of phytochemicals is isolation. Column and thin-layer chromatography have been considered widely employed techniques for this purpose [82,83,84]. The adsorption-based separation in the column results in the isolation of different kinds of phytochemicals based on their polarity and affinities toward stationary and mobile phases. In addition, the purification process can be accelerated using developed instruments such as HPLC.

For the determination of crude proteins in extract, the macro Kjeldahl method is employed. Furthermore, inductively coupled plasma optical emission spectrometry (ICP-OES) is employed for the determination of mineral content in the sample. The thermal desorption method has been employed by researchers for flavor and fragrance profiling. For the determination of alkaloids and tannins, the extract is treated with Hager’s reagent and a 5% ferric chloride reagent, respectively. Folin Ciocalteu’s reagent and aluminum chloride colorimetric reagents are employed for the determination of total phenolic content and total flavonoids, respectively [28,85]. The important biochemicals and other food components reported to be present in the *C. album* are summarized in tabulated form in Table 1.

For identification and fingerprinting of secondary metabolites present in the sample, sophisticated and hyphenated techniques, including HPTLC, HPLC, GC, LC-MS, GC-MS, ICP-OES, GC-NMR could be employed [86,87,88].

**Table 1:** Important food components and biochemicals reported in different extracts of *C. album* by scientists.

Plant Part	Sample Processing	Extraction Technique	Solvents Employed in Extraction	Reported Phytochemicals	Analytical Method/Technique (s) Employed for Detetction	Reference
Aerial parts	Shade drying, pulverizing, defatting	Soxhlet extraction, cold maceration	Soxhlation: Ethyl acetate, acetone, and methanol Maceration: 50% Methanol	Flavonoid (7.335 mg/g) (Quercetin)	UV–visible spectroscopy (UV–Vis), Infrared spectroscopy (IR), Nuclear magnetic resonance spectroscopy (NMR), and Mass spectrometry (MS) Aluminium chloride method (for flavonoid)	[86]
	Washing, drying, grinding	Cold maceration	Maceration: Acetone	Xanthophylls (331 mg/100 g dry wt): neoxanthin, violaxanthin, lutein (11.7 to 185 mg/100 g dry wt), zeaxanthin, Provitamin A: 120 mg/100 g dry wt)	High-performance liquid chromatography—Photodiode Array (HPLC-PDA), LC-MS	[72]
	Size reduction	Cold maceration, centrifugation	Maceration: Acetone	Apocarotenoids, chenoalbicin (0.02% yield)	HPLC, UV–Vis, Column chromatography (CC), NMR, High-resolution Electrospray Ionization Mass Spectrometry (HREIMS)	[89]
	Crushing, defatting	Solvent extraction	Acetone, water, petroleum ether	β-carotene (0.19–5.91 mg/100 g fresh wt)	Column Chromatography	[90]
	Washing, drying, grinding, airtight storage	Solvent extraction	0.05 M Phosphate buffer, 80% aqueous methanol	Total phenols (304.98 GAE) Saponin (0.027–0.867 g/100 g) Phytic acid (268.33 mg/100 g) Alkaloid (1.27–1.67 mg/100 g) Flavonoid (220.0–406.67 mg/100 g) Oxalate (518.45 mg/100 g)	Folin–Ciocalteu reaction (Phenols), Prothrombin time (saponins), colorimetric method (phytate), UV and MS (flavonoids) Chromatography (alkaloids)	[80]
	Washing, shade drying, grinding, airtight storage	Hydro-distillation	Water	Essential oil (0.466% *v/w*) (mainly containing α-pinene, β-pinene, linalool, α-terpineol, ascaridole, carvacrol, phytane, linolenic acid, diosgenin)	Gas chromatography (GC)-MS	[22]
	Pulverization	Hydro-distillation	Water	Essential oil (0.64% *v/w*) [α-thujene, α-pinene (7%), ascaridole (15.5%), myrcene, sabinene, p-cymene 40.9%, limonene, camphene, carvacrol, elemicin, neral, citronellal, borneol, γ-terpineneol (6.2%)]	Gas chromatography—Flame ionization detector (GC-FID) and GC-MS	[18]
	Drying, coarse powdering	Maceration	Methanol, chloroform, n-hexane, petroleum ether, acetone	Alkaloids, amino acids, cardiac glycosides, anthraquinone, flavonoids, steroids, starch	UV–Vis	[17]
	Air drying, pulverization	Soxhlation Maceration	Soxhlation: Chloroform, acetone, ethyl acetate, methanol Maceration: 50% methanol	Alkaloids, carbohydrates, amino acids, flavonoids, saponins, tannin, sterol, terpenoids	Thin-layer chromatography (TLC)	[14]
	Shade drying, grinding	Soxhlation	Hexane, ethyl acetate Soxhlation: ethanol	Astragalin (50.75% of total extract)	CC, TLC, High-Performance (HP)-TLC, HPLC, UV, Fourier Transform (FT)-IR, NMR	[91]
	Shade drying, grinding	Solvent extraction	Ethanol, water	Carbohydrates, protein, alkaloid, tannin, saponin, and flavonoid	HPTLC, Fluorescence spectroscopy	[92]
	Cleaning	Microwave-assisted extraction	Petroleum ether, ethyl acetate, methanol, hydroalcoholic, and aqueous solvent	Alkaloids (1.77 to 2.80 mg/g equivalent of atropine), flavonoids (1.72 to 3.81 mg/g equivalent of quercetin), saponins (3.05 to 3.22 mg/g equivalent of diosgenin), total phenols (1.77 to 2.94 mg/g equivalent of gallic acid)	UV, Folin–Ciocalteu reaction (Phenols), Aluminium chloride method (for flavonoid), Vanillin reagent method (saponins), Bromocresol green reaction method (alkaloids)	[93]
Aerial parts and roots	Air drying, grinding	Maceration	n-hexane, acetone, methanol	Total phenolics 64.37 µg PEs/mg of the extract (protocatechuic acid, rutin, hesperidin (9769.13 ± 158.26 μg/g extract), rutin (2935.19 ± 39.92 μg/g extract), apigetrin, quercetin, astragalin, apigenin, and luteolin), Flavonoids (126.67 µg QEs/mg of extract) Fatty acids (mainly with myristic acid 18.26% and cis-10-pentadecanoic acid 15.93%)	Folin–Ciocalteu reaction (Phenols), UV–Vis (flavonoids), LC-MS/MS, GC-MS	[88]
Seeds/Grains	Powdering	Solvent extraction	Water	Oleanolic acid, glucose, glucuronic acid,	Ion exchange (IEX) CC, TLC, NMR	[94]
	Cleaned, dried, milled	Solvent extraction	Sodium hydroxide, water	Carbohydrates, protein, fiber, fat	Colorimetry	[95]
	Air drying, grinding	Soxhlation Infusion	Soxhlation: Benzine Infusion: Chloroform, methanol	Lipids (5.8 to 8.9%) (neutral, glycolipids, phospholipids, fatty acids), carotenoids (6.61 mg/100 g) Fatty acids (oleic acid 37.9%, linoleic acid, 26.1%, palmitic acid 17.4%, lignoceric acid 1.1%)	CC, TLC, GC, FTIR	[96]

## 6. Impact of Processing Methods on Bioactive Composition and Stability of *C. album*

Green leafy vegetables, as a rich source of minerals, vitamins, and dietary fibers, are ubiquitously consumed in India. Leafy greens undergo several physical and chemical processing treatments during the cooking process, which affects the nutritional and functional properties of leaves. *C. album* leaves are generally stewed (largely replaced by pressure cooking) or stir-fried before consumption. Additionally, the leaves are mixed with other leaves, such as mustard and spinach, while cooking saag (an Indian dish). Different processing methods employed in the course of cooking have been reported to out-turn in the loss of nutrients [90]. Rather than nutritional losses, the taste and convenience of preparation are practically preferred while cooking at home. The effects of different processing techniques on the bioactive composition are discussed below.

### 6.1. Cooking and Thermal Effects

Cooking includes various methods and their combinations which are used to prepare food to consume. β-carotene, an important phytoconstituent, with potent antioxidant and cardioprotective actions, in several vegetables has been reported to decrease by 5% to 78% during cooking by different methods [97,98]. Pressure cooking of leaves has resulted in the loss of β-carotene content of the leaves. Surprisingly, the saag prepared by *C. album* leaves in the traditional Punjabi style was found to increase the β-carotene content (32 mg/100 g), probably due to added mustard leaves [99]. The method of preparation involved initial pressure cooking, blending, maintenance of consistency using maize flour, and mixing the above-prepared mixture while serving with a separately fried mixture of onion, garlic, and ginger. In another study, the effect of stir-frying and pressure cooking was determined. Results revealed the superiority of stir-frying (average loss, iron 13.48% and β-carotene 10.2%) with respect to pressure cooking (average loss, iron 32.57% and β-carotene 19.44%) method [90].

In addition, thermal processing has resulted in the maximum unfolding of native protein structure and improvement in the thermal stability of album protein isolates (APIs). The unfolding of the native protein structure can be correlated to the improvement of its in vivo digestibility. The lysine content of the protein becomes lowered due to heating. The improvement in in vitro digestibility and nutritional and physicochemical properties of APIs suggested the positive impact of heat treatment [100].

The effect of the processing method on the content of antinutritional factors present in the plant should be considered. The process of boiling for 2 min notedly reduced the soluble oxalate content locked in leaves from 33% to 22% by leaching, while wok-frying (170 °C for 2 min) resulted in an increased in its content (79%). However, the availability of soluble calcium oxalate, which could be harmful to human kidneys, is significantly decreased by wok-frying. Moreover, the pesto and juice prepared from the leaves and added vegetables reported lower content of soluble oxalates due to their removal after binding with fiber [12].

To investigate the effect of roasting and germination on the carbohydrates and anti-nutritional constituents, the *C. album* seeds were roasted (in a preheated cauldron) and made to germinate (72 h). The processing increased total reducing sugar content, whereas starch and non-reducing sugar content decreased. The reduction in total phenol and tannin content was found to be decreased significantly by roasting compared to germination. In contrast to germination, roasting resulted in decreased flavonoid content of the seeds [101]. Interestingly, the processed flour was found to have increased mineral (Na, Cu, Zn), dietary fiber, and predominant fatty acid (linoleic, oleic, and palmitic) contents when germinated seeds were employed instead of raw seeds. Furthermore, the phytochemical content of flour was also found to be increased [25].

In conclusion, thermal processes, including boiling and cooking with the traditional Punjabi style, positively enhance nutrition, while roasting, wok-frying, and pressure cooking negatively influence the nutritional content. On the other hand, flour prepared from germinated seeds and leaf juice is known to be rich in phytochemicals and decreased oxalate content.

Table 2 presents important nutrients and minerals found in *C. album* and the impact of different processing/cooking methods on their content.

### 6.2. Blanching and Drying

In an attempt to investigate the effects of blanching and drying of leaves, the leaves were made to undergo the same treatments. Blanching decreased while drying increased the total content of phenolics and flavonoids. The antioxidant activity was also increased by drying [102]. Additionally, the leaves were subjected to sunlight for 8–10 h, a hot air oven for 5 h, and microwave (4 min) drying to investigate the effect of drying on nutrient composition. Results concluded that hot air oven drying was the optimum, showing acceptable retention of carbohydrates, proteins, and antioxidant activity similar to that dried by the traditional technique [103].

Despite the loss of nutrients, the bioavailability of some nutrients becomes enhanced due to cooking. The bioavailability of calcium and zinc is reported to increase by blanching for 5, 10, and 15 min, as evidenced by significantly increased HCl extractability [104], while the cooking of *C. album* leaves increases the iron bioavailability from 12.8% to 21.2% [105]. The increase in mineral bioavailability is suggested to be caused due to removal of antinutrients like phytic acid and oxalic acid during blanching and cooking.

### 6.3. Dehydration

Leaves are sometimes dehydrated to store them for future use due to concentrated micronutrients. Leaves are dehydrated by initial chemical (0.1% magnesium oxide, 0.1% sodium bicarbonate, and 0.5% potassium metabisulphite) treatment followed by steam blanching for 5 min, and then dried in an oven (60 °C for 12 h) and powdered. Dehydration was found to cause only a small effect on the proximate mineral and antinutrient content of leaves [106].

### 6.4. Ultrasound Treatment

The treatment of food products with ultrasound causes changes in the native form of proteins which are advantageous vis-a-vis protein functionality. Ultrasound treatment causes a reduction in the size of protein isolates, increased surface hydrophobicity, and hence some degree of the unfolding of the protein structure, which alters their functional characteristics [107,108]. High-intensity ultrasound treatment of protein isolates from *C. album* results in significantly increased solubility, whiteness index, foam stability, foaming capacity, and molecular weight loss. Moreover, the increase in the time of ultrasound treatment results in decreased denaturation temperature, which might occur due to the breaking of molecular bonds and hence structural and conformational changes [109]. The denaturation temperature of protein from 84.56 to 75.90 °C was found to be decreased with increased duration of ultrasound treatment from 5 to 25 min. In *C. album* APIs-based food packaging film, high-intensity ultrasound treatment with different lysozyme concentrations improves physicochemical, functional, and mechanical characteristics [110].

**Table 2:** Important nutrients and minerals found in *C. album* and the impact of different processing/cooking methods on their content.

S. No.	Processing/Storage Method	Time Period	Impact on Nutrient and Mineral Content (and Method Employed for Analysis)										Reference(s)
			Ascorbic Acid	β Carotene	Oxalic Acid/	Phytic Acid	Polyphenols/TPC	Dietary Fiber	PUFA	Calcium	Iron	Zinc	
1.	Refrigeration without packaging at 5 °C	24 h	Decreased by 4.40% (T)	No considerable loss (CM/SM)	Decreased by 3.76% (T)	Decreased by 0.22% (CM/SM)	Decreased by 2.80% (CM/SM)			-	-	-	[111,112]
		48 h	Decreased by 7.06% (T)	Decreased by 1.75% (CM/SM)	Decreased by 3.76% (T)	Decreased by 0.22% (CM/SM)	Decreased by 4.26% (CM/SM)			-	-	-	[111,112]
2.	Refrigerated in polyethene bags at 5 °C	24 h	Decreased by 2.03% (T)	No considerable loss (CM/SM)	Decreased by 0.88% (T)	Decreased by 0.22% (CM/SM)	Decreased by 1.19% (CM/SM)			-	-	-	[111,112]
		48 h	Decreased by 5.65% (T)	Decreased by 0.77% (CM/SM)	Decreased by 4.02% (T)	No considerable loss (CM/SM)	Decreased by 2.28% (CM/SM)			-	-	-	[111,112]
3.	Stored in polyethene bags at 30 °C	24 h	Decreased by 45.76% (T)	Decreased by 1.87% (CM/SM)	Decreased by 0.97% (T)	Decreased by 0.08% (CM/SM)	Decreased by 2.61% (CM/SM)			-	-	-	[111,112]
		48 h	Decreased by 66.90% (T)	Decreased by 2.84% (CM/SM)	Decreased by 3.76% (T)	No considerable loss (CM/SM)	Decreased by 3.55% (CM/SM)			-	-	-	[111,112]
4.	Sun Drying	10 h	Decreased by 88.25% (T)	Decreased by 48.50% (CM/SM)	- *	- *	Decreased by 0.43% (CM/SM)			-	-	-	[111,112]
		Till 6–7% moisture content	Decreased by 29.73% (DRM)	Increased by 758.38% (CC)	Decreased by 29.02% (T)	Decreased by 42.61% (CM/SM)	Decreased by 16.13%			Increased to 459.29% (AAS)	Increased to 536.86% (AAS)	Increased to 322% (AAS)	[113]
5.	Oven drying at 60 to 65 °C	10 to 12 hr	Decreased by 87.40% (T)	Decreased by 16.03% (CM/SM)	No considerable loss (T)	No considerable loss (CM/SM)	- *						[111,112]
		Till 6–7% moisture content	Decreased by 43.24% (DRM)	Increased by 842.55% (CC)	Decreased by 47.50% (T)	Decreased by 53.54% (CM/SM)	Decreased by 28.31%			Increased to 523.15% (AAS)	Increased to 639.27% (AAS)	Increased to 368% (AAS)	[113]
6.	Shade drying	Till 6–7% moisture content	Decreased by 8.10% (DRM)	Increased to 822.94% (CC)	Decreased by 19.07% (T)	Decreased by 28.508% (CM/SM)	Decreased by 35.38%			Increased to 484.91% (AAS)	Increased to 589.15% (AAS)	Increased to 396% (AAS)	[113]
7.	Solar drying	Till 6–7% moisture content	Decreased by 13.51% (DRM)	Increased by 682.61% (CC)	Decreased by 22.33% (T)	Decreased by 44.69% (CM/SM)	Decreased by 22.54%			Increased to 385.26% (AAS)	Increased to 522.16% (AAS)	Increased to 298% (AAS)	[113]
8.	Blanching	5 min	Decreased by 56.20% (T)	Decreased by 9.82% (CM/SM)	Decreased by 27.69% (T)	Decreased by 1.36% (CM/SM)	Decreased by 3.60% (CM/SM)			-	-	-	[111,112]
		10 min	Decreased by 71.38% (T)	Decreased by 20.17% (CM/SM)	Decreased by 21.97% (T)	Decreased by 1.89% (CM/SM)	Decreased by 14.63% (CM/SM)			-	-	-	[111,112]
		15 min	Decreased by 95.06% (T)	Decreased by 28.49% (CM/SM)	Decreased by 35.38% (T)	Decreased by 2.16% (CM/SM)	Decreased by 23.21% (CM/SM)			-	-	-	[111,112]
9.	Open-pan cooking	30 min	Decreased by 96.31% (T)	Decreased by 2.48% (CM/SM)	Decreased by 22.71% (T)	Decreased by 0.06% (CM/SM)	Decreased by 1.55% (CM/SM)			-	-	-	[111,112]
10	Pressure cooking	10 min	Decreased by 89.58% (T)	Decreased by 1.34% (CM/SM)	Decreased by 26.03% (T)	Decreased by 0.08% (CM/SM)	Decreased by 0.76% (CM/SM)			-	-	-	[111,112]
		10 min	-	Decreased by 19.44% (CC)	-	-	-			Decreased by 0.81 to 9.43% (AAS)	Decreased by 28.79 to 36.34% (AAS)	Decreased by 5.47 to 5.63% (AAS)	[90]
11.	Stir frying	15 min	-	Decreased by 10.2% (CC)	-	-	-			Decreased by 2.68 to 8.45% (AAS)	Decreased by 13.08 to 13.88% (AAS)	Decreased by 4.93 to 7.81% (AAS)	[90]
12.	Germination followed by milling to flour	-	-	-	-	-	Increased to 234.43% (CM/SM)			-	-	-	[114]
13.	Germination followed by milling to flour							Increased to 108.76% (DM)	Increased by 1.24% (GC-FID)	Decreased by 15.54% (AAS)	Decreased by 64.7% (AAS)	Increased to 100.82% (AAS)	[25]

T—Titration method, CM/SM—colorimetry/spectrophotometry, CC—column chromatography, DRM—Dye reduction method, TPC—Total phenolic content, AAS—Atomic absorption spectroscopy, DM—Digestion method, GC-FID—Gas chromatography–flame ionization detection. * represents the value used as control.

## 7. Functional Activities of *C. album*

### 7.1. Antimicrobial Activities

Antimicrobial activity of different molecules is executed by several modes, including disruption/inhibition of cell wall synthesis and protein synthesis, and by binding to different functional units and inhibiting their functions. Interestingly, *C. album* has been reported for its valuable activity against bacteria, fungi, and nematodes. The antimicrobial activity of the *C. album* is discussed below.

#### 7.1.1. Antibacterial Activity

The antibacterial activity of oil obtained by hydro-distillation of *C. album* leaves against Gram-positive and Gram-negative strains has been investigated [22]. Agar well diffusion, agar disc diffusion and microdilution assays revealed significant antibacterial activity against MDR bacterial strains with inhibition zones ranging from 7.0 ± 0.0 mm to 16.0 ± 6.6 mm (in the disc diffusion method) and from 7.0 ± 0.6 mm to 15.0 ± 1.0 mm (in the well diffusion method). Among the tested strains, oil was found to be most effective against *Escherichia coli* (MIC 1.25 mg/mL and MBC 2.5 mg/mL), *Shigella dysenteriae* (MIC 0.62 mg/mL and MBC 1.25 mg/mL), *Shigella sonnei* (MIC 1.25 mg/mL and MBC 2.5 mg/mL), *Salmonella typhimurium* (MIC 0.31 mg/mL and MBC 0.62 mg/mL), and *Staphylococcus aureus* (MIC 1.25 mg/mL and MBC 2.5 mg/mL).

In another study, the water extract of *C. album* exhibited significant anti-bacterial action against Gram-positive (*Bacillus cereus* MIC 0.5 mg/mL, *Staphylococcus epidermidis* MIC 0.5 mg/mL, *Staphylococcus aureus* MIC 1.0 mg/mL, *Micrococcus cristinae* MIC 0.5 mg/mL, and *Streptococcus pyogens* MIC 0.5 mg/mL) and Gram-negative (*E. coli* MIC 1.0 mg/mL, *Salmonella pooni* MIC 1.0 mg/mL, and *Serratia marcescens* MIC 1.0 mg/mL) strains [115].

Additionally, Korcan and co-authors investigated the antibacterial activity of methanolic extract and reported maximum activity against *Bacillus subtilis* (13 mm zone of inhibition at 100 µg/mL), and the activity was found to be increased with increasing concentration of extract [116]. Lone et al. reported the maximum inhibiting activity of the methanolic extract against *S. aureus* with an inhibition zone of 28 ± 0.14 mm and a mild effect against *E. coli* among the tested organisms [117]. Interestingly, Umar and colleagues employed the leaf extract as a reducing agent in the development of reduced graphene oxide nanoparticles displaying antimicrobial activities. The significant antibacterial activity of the plant warrants its potential as a potent antibacterial agent [118]. However, further research is needed to isolate and identify the specific molecules responsible for the action.

#### 7.1.2. Antifungal Activity

The antifungal potential of *C. album* against various pathogenic phyto-fungi has attracted scientific interest in recent years. Alkooranee et al. investigated the antifungal potential of *C. album* roots and leaves against phytopathogenic fungi, including *Alternaria alternate*, *Fusarium solani*, *Pythium aphanidermatum*, *Rhizoctonia solani*, and *Sclerotinia sclerotium*. The water extract of leaves and roots was reported to have a significant mycelial growth reduction effect [119]. Furthermore, Sherazi [120] explored the antifungal activity of *C. album* for the management of *Ascochyta rabiei*, which are implicated in chickpea blight. Among different fractions of methanolic extract of *C. album* leaves, the n-hexane fraction exhibited the highest antifungal potential. In addition to the above, Javid and Rauf utilized the methanolic leaf extract for controlling the basal rot disease (caused by *Fusarium oxysporum*) of onion [121]. The chloroform fraction of methanolic extract exhibited the maximum antifungal activity by reducing 96–100% of fungal biomass. Inflorescence extract possesses the highest antifungal activity against *F. oxysporum*, and a possible management of the problem was achieved by employing methanolic inflorescence extract as a natural fungicide [122]. The antifungal potential of plant roots against *Sclerotium rolfsii*, soil-borne phytopathogenic fungi has been investigated. The methanolic extract of roots was reported to significantly reduce fungal biomass. Furthermore, the abundant compound in the extract, as identified by GC-MS, was found to be mono(2-ethylhexyl)ester of 1,2-benzenedicarboxylic acid [74].

### 7.2. Anthelmintic Activity

The current epidemiology, unavailability of vaccines for intestinal infections, and the resistance to chemotherapy have made it compelling to discover and develop novel anthelmintic drugs. Medicinal plants, which are continuously the source of novel medicinally useful molecules, have attracted scientific interest for the purpose. Further, Peachey et al. [123] investigated the anthelmintic potential of *C. album* against cyathostomins, a most important gastrointestinal nematode infecting equids. Plant extracts were prepared by drying, milling, macerating with methanol, and vacuum evaporation. Larval migration inhibition test and egg hatch test revealed the significant anthelmintic activity of *C. album*, with the lowest value of EC_50_ at 2.3 mg/mL. Lone et al. [117] reported dose- and time-dependent anthelmintic activity against *Haemonchus contortus*. Worm mobility inhibition assay and fecal egg count reduction assays were used to determine in vitro and in vivo anthelmintic activity. Methanolic extract possessed more significant anthelmintic activity than aqueous extract. In contrast, it has been reported that the aqueous extract is better in terms of anti-parasitic activity against *H. contortus* [85]. The anthelmintic potential of *C. album* extracts against adult *Eisenia fetida*, an Indian earthworm, has been investigated [124]. Among all extracts, ethyl acetate was reported to exhibit highly significant anthelmintic activity at a 10 mg/mL concentration by causing paralysis and fatality of the earthworms, with paralysis and death time reported to be 10.08 ± 1.11 and 65.28 ± 2.09, respectively. Furthermore, the reversible paralysis of body wall muscle was caused due to the GABA-mimetic action of the extract. Whole-plant extract of *C. album* was also reported to possess ovicidal efficacy against GI nematodes in goats with ED_50_ and ED_90_ values of methanolic, ethylacetate and chloroform extracts at 3.86 and 7.14, 2.73 and 8.31, 4.41 and 20.11 mg/mL, respectively [125]. The anthelmintic and other biological effects reported in *C. album* are covered in Table 3**.**

### 7.3. Hepatoprotective Activity

Infections, increased alcohol consumption, anemia, malnutrition, and availability of hepatotoxic drugs over the counter have been implicated as the most common reasons behind liver diseases [137]. The conventionally used drugs for the treatment may lead to serious adverse effects. The use of natural remedies prepared from medicinal plants could be promising. In such context, the hepatoprotective potential of *C. album* has been intensively explored by researchers in recent years. In vitro hepatoprotective potential of aerial parts of *C. album* in Hep G2 cells was reported; compounds isolated from extracts including nemanolone D, nemanolone E, and substituted 5,7-dimethoxy-cyclohepta-furan-6-one derivatives showed notable activity to lower AST and ALT levels in Hep G2 cells treated with H_2_O_2_ and hepatoprotective potential against paracetamol-induced liver damage [131,138]. Moving further, biochemical marker analysis and histopathological studies revealed the hepatoprotective action of aqueous and alcoholic extract as evinced by restored levels of alkaline phosphatase, bilirubin content, and serum transaminases, and reversal of liver damage induced by the toxin. Moreover, the methanolic extract of *C. album* has been reported to protect the liver against ethanol-induced liver damage [139]. The liver protection potential of the plant is extended to CCl4-induced liver damage. The extracts of powdered plant material were found to alleviate the CCl4-induced elevated levels of ALT, AST, bilirubin, and total cholesterol. The methanolic extract was found to exhibit the significant hepatoprotective activity the most [140,141,142].

### 7.4. Antioxidant Activity

The general in-built antioxidant mechanism of the human body, which ought to equipoise between reactive oxygen species (ROS) production and antioxidant activity, is often disturbed in long lifetime, increased oxygen consumption, and production of reactive nitrogen species. The oxidative stress generated in this was leads to the pathogenic development of neurodegenerative diseases, diabetes, cancer, vascular diseases, kidney diseases, pulmonary diseases, and aging. Plant polyphenols, including flavonoids and non-flavonoids, possess significant antioxidant activity to combat radical-initiated oxidative damage [143,144,145,146,147,148,149].

The mechanism of antioxidation involves the scavenging of free radicals and/or inhibition of the production of reactive oxygen species [150,151]. *C. album*, due to the presence of a considerable amount of polyphenols, exhibits strong antioxidant activity. The antioxidant activity of aqueous and alcoholic extract of *C. album* was assessed by DPPH (2,2-diphenyl-1-picryl-hydrazyl-hydrate) assay, superoxide anion radical scavenging activity-riboflavin photo-oxidation method, hydroxyl-scavenging activity-deoxyribose assay, and lipid peroxidation method. DPPH assay revealed the aqueous extract to possess the highest percentage (96%) of free radical inhibition while the riboflavin photo-oxidation method and hydroxyl scavenging method evinced methanolic extract to exhibit the highest and most significant antioxidant activity [117]. Moreover, superior DPPH free radical scavenging activity has also been reported [152]. Researchers employed NBT (nitroblue tetrazolium reduction test) and ABTS (2,2′-azino-bis(3-ethylbenzothiazoline-6-sulfonic acid) radical scavenging activity methods in extra to assess the antioxidant activity of *C. album* seed extracts. The superior DPPH free radical scavenging of aqueous extracts may occur due to the presence of more hydrogen donors to scavenge the free radical [117]. The results of the Ferric reducing antioxidant power (FRAP) assay, in addition to the ABTS assay, have corroborated the antioxidant activity of *C. album* [28]. Table 4 lists some recent reports on the antioxidant activity of *C. album*.

Furthermore, the plant extract has also been reported to possess the potential to protect the drug-induced toxicities. The ameliorative effect of *C. album* in cyclophosphamide-induced oxidative stress and hematologic toxicity has been evinced [153], where the lymphocyte and hemoglobin content was found to be significantly increased on *C. album* treatment.

**Table 4:** Table enlisting recent reports on antioxidant activity of various parts of *C. album*.

S. No	Antioxidant Assay Method	Plant Part	Type of Extract	Findings	Reference
1.	DPPH assay	Whole-plant powder	Methanolic (MT) and aqueous	At 300 µg/mL Aqueous extract inhibited 96%; MT inhibited 73%	[117]
		Seed	Chloroform (CF), ethyl acetate (EA), acetone (AT) and MT extracts	At 200 µg/mL MT inhibited 87.83%; AT—84.55% EA—86.41% CF—80.44%	[152]
		Aerial parts	Hexane (HE), EA, CF extracts	IC50 (µg/mL): Hexane—>1000, EA—140, CF—435	[154]
		Seed	MT extracts	At 0.1 mg/mL, MT inhibited 74%	[155]
2.	Superoxide anion radical scavenging activity riboflavin photo-oxidation method nitroblue tetrazolium assay	Whole-plant powder	MT and aqueous	At 300 µg/mL, aqueous extract inhibited 74% while MT inhibited 85%	[117]
		Seed	Petroleum Ether (PE), CF, EA, AT, and MT extracts	At 200 µg/mL, MT inhibited 66.79%, AT—60.35%, EA—68.29%, CF—64.04%	[152]
3.	Hydroxyl scavenging activity- deoxyribose assay	Whole-plant powder	MT and aqueous	At 300 µg/mL, Aqueous extract inhibited 83% while MT inhibited 94%	[117]
4.	Modified thiobarbituric acid reactive species assay	Whole-plant powder	MT and aqueous	At 300 µg/mL, Aqueous extract inhibited 86% while MT inhibited 78%	[117]
5.	H_2_O_2_ scavenging assay	Seed	PE, CF, EA, AT, and MT extracts	At 200 µg/mL, MT inhibited 87.67%, AT—75.85%, EA—78.86%, CF—85.57%	[152]
6.	ABTS (2,2′-azino-bis(3-ethylbenzothiazoline-6-sulphonic acid) radical scavenging assay	Seed	PE, CF, EA, AT, and MT extracts	At 200 µg/mL, MT inhibited 85.70%, AT—84.77%, EA—87.17%, CF—88.22%	[152]
7.	β-Carotene bleaching test	Aerial parts	Hexane, EA, CF extracts	IC50 (µg/mL) at 60 min: Hexane—>100, EA—38.03, CF—>100	[154]

### 7.5. Anticancer Activity

The continuous hunt for novel anticancer drugs and previously discovered anticancer molecules from plants has led to increased scientific attention towards plant extracts and the phytochemicals present in them. Phytochemicals, including taxanes, Catharanthus alkaloids, geniposide and derivatives, and other compounds such as artesunate, colchicine, and roscovitine possess the significant anticancer potential to be used in the management of cancer [156].

The anticancer potential of commonly consumed plant *C. album* has been assiduously scrutinized by researchers across the globe. Rana et al. investigated the in vivo anticancer potential of *C. album* leaves against Ehlrich ascites carcinoma (EHC) cells in Swiss albino mice. Results have revealed the statistically significant cell growth inhibition of 30.60% and 41.80% at the concentration of 200 mg/kg and 400 mg/kg of *C. album* extract, respectively. Moreover, the treatment was found to reinstate all the biochemical parameters, including hemoglobin content, red blood cell (RBC), and white blood cell (WBC) count in the mice. The inhibition of cell growth, a decrease in tumor weight, increase in mean survival time, and induction of apoptosis were all concluded to be contributors to the anticancer effect. Plant-sourced lectins are known to instigate apoptosis and autophagy of cancer cells [157]. In a study, lectin (175 µg/mL) isolated from *C. album* seeds showed strong anti-cancerous potential in hepatoma HepG2 cells as evinced by significantly decreased transcription levels of AFP and GPC3 levels by 90% and 89%, respectively [158].

Interestingly, Umar et al., 2020, formulated the reduced graphene oxide nanoparticles using an extract of *C. album* as a reducing agent. The anticancer activity against MCF-7 cells of the developed nanoparticles suggested a new approach for the treatment of breast cancer [118]. Moreover, tropolone-based compounds, which included two new tropolones and three known tropolone derivatives (lactarotropone, 2,9-epoxylactarotropone, and 6-lactaradien-5-oic acid γ-lactone) obtained from the aerial parts of the plant have been reported to exhibit notable in vitro anticancer activity against human tumor cell lines including HGC-27 (stomach cancer cells), MDA-MB-231 (breast cancer cells), A-549 (lung cancer cells), HCT-116 (colon cancer cells), and A2780 (ovarian cancer cells) with IC_50_ values ranging from 0.5 ± 0.2 to 15.5 ± 2.7 μM [131]. However, in vivo and clinical studies are needed to validate the anti-cancer activity of the obtained derivatives.

### 7.6. Other Activities

**Figure 2:**
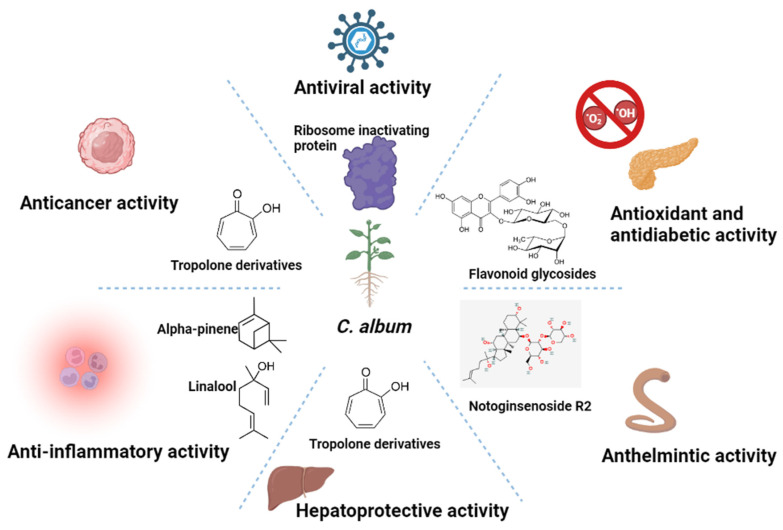
Figure representing important chemical components and corresponding biological activities of *C. album*.

Type 2 diabetes, one of the major endocrine disorders, is characterized by impaired insulin secretion and/or impaired action of insulin at the cellular level. Some plants are known and ethnobotanically used in several parts of the world for their potent antidiabetic activity, *C. album* among them [159]. A study emphasized the antidiabetic potential of methanolic extract of the plant in male Wistar albino rat models. Treatment with a high dose of *C. album* extract (500 mg/kg body weight p.o) resulted in the maximum decrease in fasting blood glucose level (139.5 ± 4.8 mg/dL, *p* < 0.01), while mild and low doses caused a slower reduction (142.2 ± 4.1 mg/dL and 148.3 ±1.5 mg/dL, *p* < 0.01 respectively) in the same at the end of the experiment (after 12 h) [127]. The flavonoid fraction is more effective than the tannin, alkaloid, and saponin fractions for lowering blood glucose levels [128]. The flavonoid fraction of *C. album* extract at doses of 250 and 500 mg/kg was reported to act by inhibition of α-amylase (IC_50_—122.18 µg/mL) in a dose-dependent manner. However, some of these doses seem to be supraphysiological and out of realistic range for human nutrition. A general layout representing the important biological activities of the plant and phytochemicals responsible is given in Figure 2.

Anti-nociception refers to the decrease in sensitivity of pre-existing painful stimuli. Results from the study revealed the anti-nociceptive activity of crude methanolic extract of *C. album* leaves [160]. The results from a study by Mushtaq and co-authors also corroborate the analgesic and anti-inflammatory activity of the plant. *C. album* resulted in the maximum (64%) inhibition of edema [161]. Interestingly, some studies have also reported the contraceptive action of the plant [162]. The anti-ulcer effect of plant extract has also been documented. It has been reported that alcohol exhibits positive effects against gastric ulcers by significantly decreasing the gastric secretion volume, free and total acidity, and ulcer index [163]. The antirheumatic potential of *C. album* aerial part extract also has been highlighted. The treatment with extract by its capacity to inhibit NF-κB significantly reduced the paw edema and normalized the level of hematological (Hb, RBC, WBC, and ESR) and biochemical (serum creatinine, total proteins, and acute-phase proteins) markers [164].

## 8. Safety and Toxicological Aspects of *C. album*

As a plant, *C. album* and its parts have been consumed in food and also utilized in the treatment of various health disorders for a long time. The millenarian use of these plants in folk medicines suggests the plant as a safe alternative for the treatment of infectious diseases and the safety profile of *C. album* seed decoction to be used as a microbicidal spermicide [117]. Methods including hemolytic index determination, dermal irritancy test, PCNA staining, TUNEL assay, and effects on local tissue and reproductive performance were employed to assess the safety standards of seed extract. The non-irritant effect of the extract on rabbit skin and rat vaginal tissues at 10-fold higher doses than its hemolytic index, along with other results of employed methods, concluded the seed extract to be a safe microbicidal spermicide. Moreover, results from the studies concluded the safety of *C. album* [165,166].

Although a high dose of phytoconstituents in concentrated form is usually not consumed and also not available in diet fractions, its safety in high doses needs to be investigated. The toxicity study has been evaluated on different extracts of *C. album*. At doses of 2000 mg/kg body wt, no notable sign of organ toxicity was observed, as evinced by histopathological analysis. However, mild toxicity in the liver, kidney, and heart was observed [128]. The acute oral toxicity studies of different doses of *C. album* alcoholic and aqueous extracts revealed the safety of extracts up to the 5000 mg/kg dose levels [138].

As already suggested in Section 6.2, blanching and drying could be a possible way towards the removal of antinutrients from the plant leaves. Considering the non-essentiality of oxalate for humans, the high water-soluble oxalate content of *C. album* leaves could be harmful to the kidneys. Interestingly, boiling has been shown to reduce the soluble oxalate content of leaves significantly [12]. However, there is a lack of literature on chronic toxicity studies of *C. album*, which present a research gap in the plant study.

## 9. Potential Food Applications

The plant is often consumed in different states of India due to its low price and nutritional importance. Blending cereals and nutritional vegetables, including spinach and mustard leaves, into the dishes prepared from *C. album* can potentially improve the nutritional value. The literature is continuously reporting on the utilization and valorization of non-conventional plants and nutrient-rich food waste [166,167,168]. The accumulated literature evinces a trendy utilization of natural raw materials from agriculture and agro-industrial waste. However, making use of *C. album* in the food science and nutraceutical sector has not received much attention from researchers.

Researchers have developed gluten-free cookies from germinated *C. album* seed flour and reported them to contain maximum antioxidant activity (23.97 g/100 g) and dietary fiber and phenolic content as that of raw *C. album* seed and wheat flour cookies [23]. Another study suggested the use of metalized polyester polyethylene to be superior to low-density polyethylene and laminated pouches for the cookie’s storage for up to 4 months under ambient conditions [24]. 

Additionally, some research studies have been carried out to evaluate the bioavailability of nutrients from different food products and bring the food potential of the plant into the spotlight. Mpreover, the bioavailability of iron from paratha and laddoo, commonly consumed foods of *C. album* leaves in India, has been determined [65]. One report also highlighted the nutritional benefits of adding dehydrated *C. album* leaves in roti [169]. Considering the nutritional importance of plant seeds, the flour of germinated seeds is used to make bread, muffins, and pancakes in China and Russia [170]. Furthermore, the utilization of GG (guar gum), XG (xanthan gum), and TG (gum tragacanth) XG at 1% concentration is advantageous for the development of gluten-free cookies from *C. album* [171]. The addition of corn starch provides lightness to the cookies. In addition to the above, the optimized cookies have been prepared and characterized as a good source of dietary fiber, essential amino acids, and minerals [172]. The market products of fresh juice made from *C. album* leaves would be highly nutritious to be consumed due to balanced amino acid, fiber, and other macronutrients.

Moreover, the *C. album* has been investigated for its positive effects on the broiler’s growth. Scientists determined fermented *C. album* to be a reliable and phytogenic feed additive which, when administered (2 to 8 g/kg) to broilers, resulted in positive effects on growth, nutrient digestibility, immunity, and meat quality. Also, the aqueous methanolic extract of *C. album* (0.1 to 1 g/kg) has shown beneficial effects on the immune response, digestive enzyme activity, and growth-promoting activity of *Cyprinus carpio* [173].

Nutraceuticals contain an interesting bioactive component possessing superlative health-boosting activities in concentrated form, and their demand has been significantly increased for human as well as veterinary use in past years due to the increasing pervasiveness of lifestyle-related diseases [174,175]. Recently, it has been highlighted that *C. album* is an important weed plant possessing nutraceutical potential [16,176]. Considering the literature published in favor of significant health-promoting activities, the plant could be utilized for nutraceutical research and development [76]. However, research to determine the in vivo bioavailability of the phytochemicals and nutrients is needed. Additionally, the research on food-based biofilms for the protection and wrapping of fruits and vegetables has added *C. album* into the list. Films prepared from *C. album* have shown antioxidative and antimicrobial properties, which evinces their possible use for fruit and vegetable packaging [110].

Despite *C. album* and similar plants possessing numerous superlative health benefits and food potential, ultra-processed and unhealthy food items available cheaply have captured the attention of retail shops over the countries, which is resulting in the gradual covering up of the population with nutrition-related non-communicable diseases. Effective measures are needed to be implemented by governments to reduce ultra-processed foods by framing policies to be employed not less than in schools and workplaces. Even though the nutritional information of *C. album* is not famous widely, its utilization is still practiced in many parts of the world. Furthermore, the successful development of nutraceuticals from *C. album* entails intensive research determining the safety, processing effects, and bioavailability of nutrients.

## 10. Discussion/Cross-Talk

A diverse spectrum of phytochemicals present in *C. album* makes the plant feature-rich in terms of nutrition and disease modification and/or treatment. The conjugation and hydroxyl groups present in several polyphenolic compounds, e.g., quercetin present in the plant, are inevitable in their antioxidant and anti-inflammatory activity. These structures are involved in the free radical scavenging and thus prevent damage at the cellular and molecular levels that translate to its antioxidant activity. Oxidative stress and inflammation are known to be destructive to several organs and thus implicated in the pathology of diseases. Polyphenolics-contributed antioxidant activity of *C. album* has shown out-turns in the form of hepatoprotective, neuroprotective, cardioprotective, and metabolic disorders. Additionally, these polyphenolic components, by acting on different checkpoints of the cell cycle and inducing pro-apoptotic gene expression, transcribe an anti-cancer effect. Despite their presence, the in vivo availability of these phytochemicals after administration is a key consideration for the manifestation of health effects. Several cooking and processing methods adversely affect the nutritional and phytochemical concentration in the plant. Cooking in the traditional Punjabi style that includes the addition of spinach and mustard leaves in addition to wheat flour positively enhances the nutritional content. In addition to the above, the anthelmintic compound ascaridole confers the potential for pharmaceutical development to the plant. However, clinical studies are needed to validate the effects in humans.

## 11. Conclusions and Future Remarks

*C. album* has remained an important dish since historic times, but its weedy presence in crops has contributed a lot to its dwindled consumption over time. In addition to enriched important nutritive components such as proteins, carbohydrates, fibers, vitamins, and minerals, the important phytochemicals present in the plant are ascaridole, carvacrol, p-cymene, cryptomeridiol, α-terpinene, chenoalbicin, and an anti-nutritional compound phytic acid. Sensorial and health-promoting activities, including antimicrobial, anti-adipogenic, antioxidant, and anti-inflammatory, are attributed to the phenolic acids present in the plant, while alkaloids contribute to the spasmolytic and anesthetic activities. Although numerous studies warrant the antimicrobial potential of the plant against bacterial and fungal species, a high concentration is needed for the execution of the activity. However, further research on the isolation and purification, as well as structural modification studies of potent antimicrobial molecules from the plant, is necessary. The phenolic content can be easily extracted from the plant using microwave- and ultrasonic-assisted extraction methods, while volatile oil and flavonoid content can be extracted using the Soxhlet extraction method. Overall, valorising of *C. album* leaves as a source of nutrition could be an advantageous step for nutraceutical development. Further, studies should focus on investigating the data on essential phytochemicals as well as the toxicology of the plant. Additionally, the physical and phytochemical properties of the plant need to be explored in depth.

## Figures and Tables

**Table 3:** Biological properties other than antioxidant activities reported in *C. album*.

S. No.	Biological Activity	Plant Part Used	Extraction/Type of Extract	Findings	Reference
1.	Anticholinesterase activity	Aerial parts and roots	Methanolic (MT), Acetone (EA), and n-Hexane (HE) extract	Butyrylcholinesterase (BchE) inhibitory activity of MT (52.64 ± 2.78%), AE (65.29 ± 1.56%), and HE (44.31 ± 2.13%)	[88]
		Fresh leaves	Leaves crushed in pestle mortar were centrifuged and supernatant used for analysis	Acetylcholinesterase inhibitory (AchE) activity (32.13%)	[126]
2.	Antidiabetic activity	Roots	MT	Decline in fasting blood glucose after a 12 h treatment with high dose (139.5 ± 4.8 mg/dL), mild dose (144.2 ± 4.1 mg/dL), and low dose (148.3 ±1.5 mg/dL)	[127]
		Aerial parts	Flavonoid fraction (CAFF), alkaloid fraction (CAAF), saponin fraction (CASF)	Alpha amylase inhibition activity in CAFF (75.66 ± 0.68), CATF (26.97 ± 0.91), CAAF (10.53 ± 1.02), and CASF (6.58 ± 0.71) at concentration of 250 µg/mL	[128]
3.	Antihyperlipidemic activity	Roots	MT	High dose MT of *C. album* normalized plasma lipid status	[127]
		Aerial parts	Hydroethanolic extract	Rats treated with cyclophosphamide along with extract 440 mg/kg b.w significantly reduced total cholesterol (53.8%), triglycerides (52.42%), and low-density lipoproteins (28.37%) compared to rats treated with cyclophosphamide alone	[129]
		Stems	MT	Rats treated with aqueous insoluble extract dissolved in PVP water mixture were reported to have marked decreased total cholesterol (53.8%), triglycerides (52.42%), and low-density lipoproteins (28.37%) compared to rats treated with cyclophosphamide alone	[130]
4.	Antiproliferative activity	Aerial parts	Ethylacetate-soluble extract fraction	IC_50_ values ranging from 0.5 ± 0.2 to 15.5 ± 2.7 µM	[131]
		Leaves	Ethylacetate Extract (EA) and MT	% Inhibition of EA and MT (100 mg/mL) against Breast adenocarcinoma estrogen-receptor-positive (MCF-7) and estrogen-receptor-negative (MDA-MB-468) cell lines was 50.40 ± 1.92, 89.09 ± 1.97 (EA), and 28.03 ± 1.97, 49.77 ± 2.01 (MT), respectively	[132]
		Seeds	MT	Desgalactotigonin and oleanolic acid-3-*O-β*-d-glucuronide found in extract-inhibited MCF-7 cells with IC_50_ value of 8.27 µM and 11.33 µM, respectively, and inhibited human topoisomerase I and II	[133]
5.	Anthelmintic activity	Aerial parts	Petroleum ether extract (PEE), EtOAcE, MTE, hydroalcoholic extract (CAHE), and aqueous extract (CAAE)	EtOAcE (10 mg/mL) was reported to have minimum time for paralysis (10.08 ± 1.11 min) and death (65.28 ± 2.09 min) of *Eisenia foetida*	[124]
		Leaves	MTE	MTE treatment for 3 h exhibited 100 ± 0.0% mortality against *Haemonchus contortus*	[134]
		Leaves and stems	MTE	Treatment with 75% and 100% MTE for 14 h resulted in 100% mortality of *Haemonchus contortus*	[85]
6.	Antimicrobial activity	Aerial parts	Ethanolic extract (EE), Chloroform extract (CE), and HE	MIC value (µg/mL) was reported to be lowest for EE against *Enterobacter aerogenes* while CE and HE shown equal results against *Bacillus subtilis*	[135]
		Leaves and roots	Aqueous extract (AE)	Against *Alternaria alternata*, *Fusarium solani*, *Rhizoctonia solani*, *Pythium aphanidermatum*, and *Sclerotinia sclerotium*, the AE (15%) of leaves showed 100%, 83.6%, 100%, 93.33%, and 91.42% mycelial growth inhibition, respectively, while AE (15%) of roots showed complete (100%) inhibition of mycelial growth	[119]
		Leaves	MT	Ethyl acetate fraction (200 mg/mL) of MT was reported to cause maximum decrease (74%) in biomass of *Sclerotium rolfsii*	[136]

## Data Availability

Not applicable.

## Abbreviations

ABTS (2,2′-azino-bis(3-ethylbenzothiazoline-6-sulfonate)), ALT (Alanine aminotransferase), API (Album protein isolates), AST (Aspartate aminotransferase), DPPH (2,2-diphenyl-1-picrazylhydrazyl), FRAP (Ferric reducing antioxidant power), FT-IR (Fourier transform infrared spectroscopy), GC (Gas chromatography), GC-MS (Gas chromatography–mass spectrometry), GC-NMR (Gas chromatography–nuclear magnetic resonance spectroscopy), GI (Gastrointestinal), HPLC (High-performance liquid chromatography), HPTLC (High-performance thin-layer chromatography), HREIMS (High-resolution electron ionization mass spectrometry), ICP-OES (Inductively coupled plasma optical emission spectrometry), IR (Infrared spectroscopy), LC-MS (Liquid chromatography–mass spectrometry), MBC (Minimum bactericidal concentration), MDR (Multi-drug resistant), MIC (Minimum inhibitory concentration), NBT (Nitroblue tetrazolium test), NCEP (Non-conventional edible plant), NMR (Nuclear magnetic resonance spectroscopy), PCNA (Proliferating cell nuclear antigen), TLC (Thin layer chromatography), TUNEL (Terminal deoxynucleotidyl transferase dUTP nick end labeling), UV (Ultraviolet spectroscopy).
